# pH-Responsive Rheological Properties and Microstructure Transition in Mixture of Anionic Gemini/Cationic Monomeric Surfactants

**DOI:** 10.3390/molecules26165013

**Published:** 2021-08-19

**Authors:** Maozhang Tian, Xi Chen, Xinyuan Zou, Yuchen Qian, Zhang Liu, Yaxun Fan

**Affiliations:** 1 State Key Laboratory of Enhanced Oil Recovery, Research Institute of Petroleum Exploration and Development, CNPC, Beijing 100083, China; mztian@petrochina.com.cn (M.T.); xchen63@petrochina.com.cn (X.C.); zouxy2016@petrochina.com.cn (X.Z.); qyc_eor@petrochina.com.cn (Y.Q.); 2 CAS Key Laboratory of Colloid, Interface Thermodynamics, Beijing National Laboratory for Molecular Sciences (BNLMS), CAS Research/Education Center for Excellence in Molecular Sciences, Institute of Chemistry, Chinese Academy of Sciences, Beijing 100190, China

**Keywords:** gemini surfactant, micellar growth, wormlike micelle, entanglement, viscoelasticity

## Abstract

Surfactant aggregates have long been considered as a tool to improve drug delivery and have been widely used in medical products. The pH-responsive aggregation behavior in anionic gemini surfactant 1,3-bis(*N*-dodecyl-*N*-propanesulfonate sodium)-propane (C_12_C_3_C_12_(SO_3_)_2_) and its mixture with a cationic monomeric surfactant cetyltrimethylammonium bromide (CTAB) have been investigated. The spherical-to-wormlike micelle transition was successfully realized in C_12_C_3_C_12_(SO_3_)_2_ through decreasing the pH, while the rheological properties were perfectly enhanced for the formation of wormlike micelles. Especially at 140 mM and pH 6.7, the mixture showed high viscoelasticity, and the maximum of the zero-shear viscosity reached 1530 Pa·s. Acting as a sulfobetaine zwitterionic gemini surfactant, the electrostatic attraction, the hydrogen bond and the short spacer of C_12_C_3_C_12_(SO_3_)_2_ molecules were all responsible for the significant micellar growth. Upon adding CTAB, the similar transition could also be realized at a low pH, and the further transformation to branched micelles occurred by adjusting the total concentration. Although the mixtures did not approach the viscosity maximum appearing in the C_12_C_3_C_12_(SO_3_)_2_ solution, CTAB addition is more favorable for viscosity enhancement in the wormlike-micelle region. The weakened charges of the headgroups in a catanionic mixed system minimizes the micellar spontaneous curvature and enhances the intermolecular hydrogen-bonding interaction between C_12_C_3_C_12_(SO_3_)_2_, facilitating the formation of a viscous solution, which would greatly induce entanglement and even the fusion of wormlike micelles, thus resulting in branched microstructures and a decline of viscosity.

## 1. Introduction

The wormlike micelles formed by surfactant solutions have been widely investigated, due to their good detergency, unique viscoelastic properties, and their large application potential [1,2,3,4,5,6,7,8,9,10]. Above a critical concentration C*, wormlike micelles can entangle into a transient network, just like flexible polymer solutions, endowing the surfactant solutions with remarkable viscoelastic properties. Under shear, these wormlike micelles can break and reform, and thus reach a dynamic equilibrium. The formation of wormlike micelles is strongly dependent on the geometry of the surfactant molecular packing. Normally, surfactants with a packing parameter of 1/3~1/2 are favorable for micellar growth [1,2,3,4,5]. For this purpose, considerable efforts have been devoted to construct wormlike micelles through controlling the inter/intramolecular interactions [6,7,8,9,10]. The variation of molecular interactions minimizes the headgroup area, induces lower spontaneous curvature and promotes micellar growth. These weak interactions are always affected by environmental factors, which provide optional ways to build switchable wormlike micelles.

Switchable wormlike micelles are one type of stimuli-responsive fluid whose reversible regulation of the rheological behaviors can be realized through introducing an external stimulus [11,12,13,14,15,16,17,18,19,20]. Based on the influence of the stimulus on these weak interactions, a series of stimuli-responsive wormlike micelles have been reported. There is vast literature [11,12,13,14,15,16] on temperature-induced aggregate formation and transition in aqueous solutions of surfactants because of their wide potential applications in many product formulations and for performing procedures. Raghavan et al. [11] investigated the influence of temperature on the aggregate transition in cetyltrimethylammonium bromide/5-methyl salicylic acid (CTAB/5mS) mixtures. The increase of viscosity upon heating can be realized through the aggregate transition from vesicles to wormlike micelles. A fraction of bound 5mS may desorb from the vesicles upon heating, leading to the decline of counter ions on the vesicles, which would increase the electrostatic repulsion between headgroups and result in the transition from vesicle to wormlike micelle. Photosensitive [17,18,19,20,21] wormlike micelles are also well documented. Sakai et al. [17] reported “photo-switchable” wormlike micelles formed in aqueous cetyltrimethylammonium bromide/sodium salicylate/4-butylazobenzene-4-(oxyethyl)trimethylammonium bromide solutions (CTAB/NaSal/AZTMA). UV-light irradiation induces the transition between trans-AZTMA and cis-AZTMA structure. As a result, the former structure promotes the wormlike micelle formation while the latter remarkably decreases the viscosity of the solution which is attributed to the disruption of the network structure of the wormlike micelles. Furthermore, a novel redox-switchable wormlike micellar system was developed based on a mixture of selenium-containing zwitterionic surfactant and sodium dodecyl sulfate [22], which reversibly and quickly responds to H_2_O_2_ and vitamin C, and shows circulatory gel/sol transition, reflecting changes in aggregate morphology from entangled wormlike micelles to vesicles.

In comparison, pH-responsive wormlike [23,24,25,26] micelles seem to be a more convenient way to construct switchable wormlike micelles, because they can be readily realized both in the laboratory and in industry. The research devotes more energy to investigating pH-induced aggregate transition in ionic surfactant systems with pH sensitive headgroups or counterions, i.e., carboxyl, phosphate, and amido. It is known that electrostatic repulsion plays a large role in maintaining the headgroup area in aggregates for ionic surfactant mixtures, while the introduced proton may bind to the pH-sensitive groups to change the electrostatic interactions between the headgroups. Huang’s group [23] reported a series of pH-sensitive viscoelastic fluids obtained by introducing a pH-responsive hydrotrope into a surfactant solution, and the pH-sensitive flowing behavior should be attributed to the aggregate transition between short cylindrical micelles and wormlike micelles. Hassan et al. [24] investigated the influence of pH on the aggregate transitions in mixtures of CTAB and anthranilic acid. At low pH conditions, the mixtures give birth to spherical micelles, and at the higher pH, the protonated anthranilic acid electrostatically binds with CTAB, in which case the headgroups are minimized and the wormlike micelles are induced. The introduced hydrogen bond through changing the pH, between COOH- and COO-, also acts on the viscosity enhancement in these pH-responsive wormlike micelles.

Gemini surfactants, constructed by two hydrophobic chains and two headgroups covalently connected by a spacer group, provide an efficient method to shorten the distance between the adjacent headgroups and facilitate the enhancement of the rheological properties and applications [27,28,29,30,31,32,33]. Zana’s group [34,35,36,37] revealed that the cationic gemini surfactant with a spacer of 2- or 3-carbon forms thread-like and entangled wormlike micelles even at low concentrations, whereas the corresponding monomeric ammonium surfactants can form only spherical micelles. Pei et al. [36] compared the rheological properties of two gemini surfactants, C_12_C_3_C_12_Br_2_ and C_12_C_3_(OH)C_12_Br_2_, and found that the anchored OH on the spacer would favor viscosity enhancement. These are attributed to the role of the intermolecular hydrogen bonding which occurs between the hydroxyl substituted spacers. Recently, it was found that pH-induced gemini-like surfactants formed by single-chain surfactant and a building block with two binding sites could also aggregate into wormlike micelles [37,38,39]. Feng et al. [37] constructed a pH-switchable wormlike micellar system by mixing *N*-erucamidopropyl-*N*,*N*-dimethylamine and maleic acid with a molar ratio of 2:1. It was assumed the neutralized amphiphilic compounds may behave as gemini surfactants that improve the rheological properties. Sakai [38] also found that mixtures of dodecanoylglutamic acid (C_12_Glu) and dodecyldimethylamine (C_12_DMA) could also aggregate into wormlike micelles at pH 5.5~6.2. They assumed the mixtures assemble into structures similar to gemini surfactants, which is favorable for the building of wormlike micelles. Our group [40] fabricated gemini-like surfactants by anionic surfactant sodium dodecyl sulfate (SDS) and cationic-charged bola-type diamines with hydrophilic or hydrophobic spacers of different lengths and found either the hydrophobic diamine with a longer spacer or the hydrophilic diamine with a shorter spacer is more beneficial for the transition from spherical micelles into rod-like or wormlike micelles.

Anionic gemini surfactant 1,3-bis(*N*-dodecyl-*N*-propanesulfonate sodium)-propane (C_12_C_3_C_12_(SO_3_)_2_) is a gemini surfactant with a 3-carbon length spacer [41]. It has been found that a cationic gemini surfactant with a 3-carbon spacer is favorable for micellar growth and the formation of wormlike micelles. However, in our previous work, C_12_C_3_C_12_(SO_3_)_2_ formed spherical micelles at low concentrations as completely deprotonated at pH 10.0. We assumed that changing the pH partly screened the electrostatic repulsion and promoted the growth of micelles, while the introduced hydrogen bond of the sulfonate group further enhanced the rheological properties both of C_12_C_3_C_12_(SO_3_)_2_ and C_12_C_3_C_12_(SO_3_)_2_/CTAB mixtures. Herein, the pH-induced aggregate transition in C_12_C_3_C_12_(SO_3_)_2_ and C_12_C_3_C_12_(SO_3_)_2_/CTAB mixtures were studied in an aqueous solution. Turbidity, rheology, dynamic light scattering (DLS) and cryogenic transmission electron microscopy (cryo-TEM) were employed to characterize the aggregate transitions and viscosity properties in the C_12_C_3_C_12_(SO_3_)_2_/CTAB mixed aqueous solution. The results further announce the superiority of gemini surfactants in building wormlike micelles and enhancing the rheological properties and give a deeper insight into the pH-manipulated aggregate transition in surfactant systems. The potential applications of these systems include viscosity modifiers, colloid stabilization and controlled drug delivery.

## 2. Materials and Methods

Materials. The anionic gemini surfactant C_12_C_3_C_12_(SO_3_)_2_ was synthesized and purified according to previous literature [36,37]. The cationic surfactant CTAB was purchased from TCI Co. with purity higher than 99% and was recrystallized before use. Water used for preparing the mixture solution in all experiments was from Milli-*Q* equipment, and the resistivity was 18.2 MΩ·cm.

Turbidity measurements. Turbidity measurements were carried out with a JASCO UV-550 spectrometer. The turbidity of C_12_C_3_C_12_(SO_3_)_2_ solutions and C_12_C_3_C_12_(SO_3_)_2_/CTAB mixtures were examined by UV absorbance at 450 nm at 25.0 ± 0.1 °C. Two cuvettes with 1 cm pathway were used. The measurement was performed starting from an alkaline solution of C_12_C_3_C_12_(SO_3_)_2_ solution, and the pH values were adjusted by adding HCl drop by drop.

Dynamic light scattering (DLS). Measurements were carried out by an LLS spectrometer (ALV/SP-125) with a multi-*τ* digital time correlator (ALV-5000). A solid-state He–Ne laser (output power of 22 mW at *λ* = 632.8 nm) was used as a light source, and the measurements were conducted at a scattering angle of 90°. The freshly prepared samples were injected into a 7 mL glass bottle through a 0.45 μm filter prior to measurements. The correlation function of the scattering data was analyzed via the CONTIN method to obtain the distribution of diffusion coefficients (*D*) of the solutes, and then the apparent equivalent hydrodynamic radius (*R*_h_) was determined using the Stokes−Einstein equation *R*_h_ = *kT*/6π*ηD*, where *k* is the Boltzmann constant, *T* is the absolute temperature, and *η* is the solvent viscosity. All the measurements were performed at 25.00 ± 0.05 °C.

Rheology measurement. The rheological properties of the mixed solutions were investigated at 25.00 ± 0.05 °C with a Thermo Haake RS300 rheometer (cone and plate geometry is 35 mm in diameter with the gap equal to 0.105 mm and the conicity equal to 2°). A solvent trap was used to avoid water evaporation. Frequency spectra were conducted in the linear viscoelastic regime of the samples determined from dynamic strain sweep measurements. For the solutions with low viscosity, a double-gap cylindrical sensor system with an outside gap of 0.30 mm, an inside gap of 0.25 mm, an outer diameter of 43 mm and an inner diameter of 41 mm was used. The linear viscoelastic region was determined by a strain sweep with the frequency fixed at 1.00 Hz. G′ and G″ were measured using frequency sweep performed at a constant stress of 0.1 (in the linear viscoelastic range) over a frequency range of 0.05–100 Hz.

Cryogenic transmission electron microscopy (Cryo-TEM). The samples were embedded in a thin layer of vitreous ice on freshly carbon-coated holey TEM grids by blotting the grids with filter paper and then plunging them into liquid ethane cooled by liquid nitrogen. Frozen hydrated specimens were imaged using an FEI Tecnai 20 electron microscope (LaB6) operated at 200 kV with the low dose mode (about 2000 e/nm^2^) and the nominal magnification of 50,000. For each specimen area, the defocus was set to 1–2 μm. Images were recorded on Kodak SO 163 films and then digitized by Nikon 9000 with a scanning step 2000 dpi corresponding to 2.54 Å/pixel.

## 3. Results and Discussion

**Figure 1:**
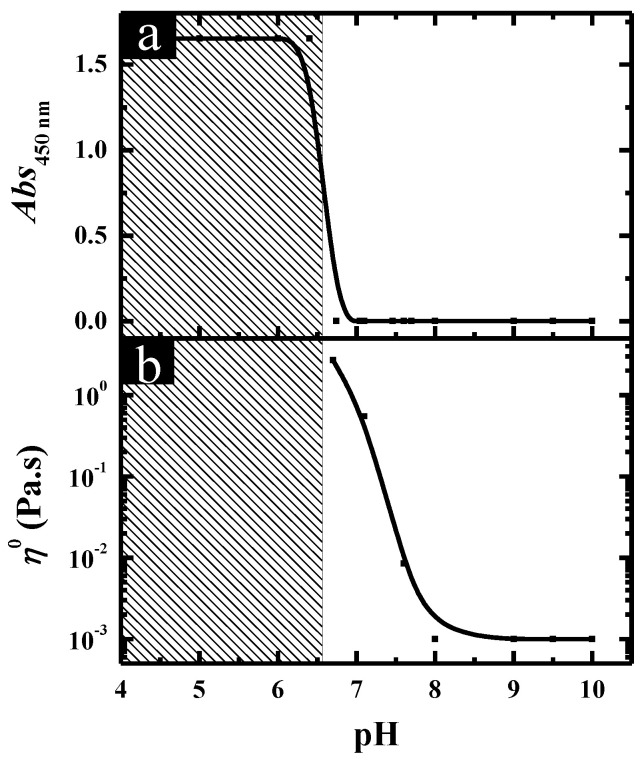
(**a**) Turbidity and (**b**) zero-shear viscosity *η*^o^ of C_12_C_3_C_12_(SO_3_)_2_ solutions plotted against pH at *C*_T_ = 20.0 mM.

The pH-induced aggregate transition in C_12_C_3_C_12_(SO_3_)_2_. Turbidity and rheology were employed to characterize the aggregate transition in C_12_C_3_C_12_(SO_3_)_2_ solutions through changing the pH, and the obtained results at total surfactant concentration (*C*_T_) of 20.0 mM are shown in Figure 1. It can be observed that upon decreasing the pH, the turbidity of the solutions remained at an extremely low value until the formation of precipitate starting from pH 6.5 (Figure 1a). Meanwhile, the variation of the zero-shear viscosity *η*^o^ for C_12_C_3_C_12_(SO_3_)_2_ solutions at different pHs, which was derived from the curves of the steady viscosity versus shear rate, is shown in Figure 1b. Correspondingly, the mixed solutions behaved as a Newtonian fluid and the viscosity remained like water above pH 7.5, following which the viscosity began to increase and reached a maximum (~5.0 Pa·s) until pH 6.5, suggesting that there is an optimal pH at which the aggregates in the solution reach the maximum growth and a maximum viscosity appears. Moreover, the solution showed shear thinning properties typically characteristic of non-Newtonian fluids in this region (6.5 < pH < 7.5).

**Figure 2:**
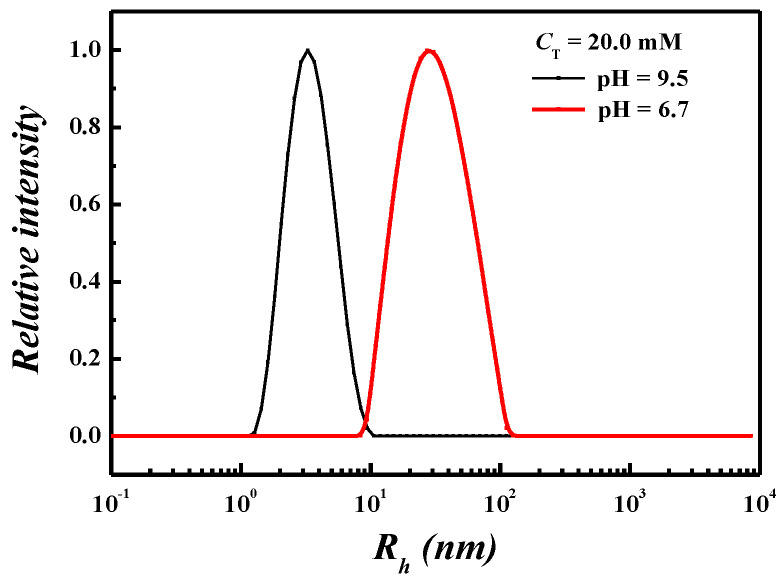
Size distributions of C_12_C_3_C_12_(SO_3_)_2_ aggregates obtained from DLS at *C*_T_ = 20.0 mM and different pH conditions.

**Figure 3:**
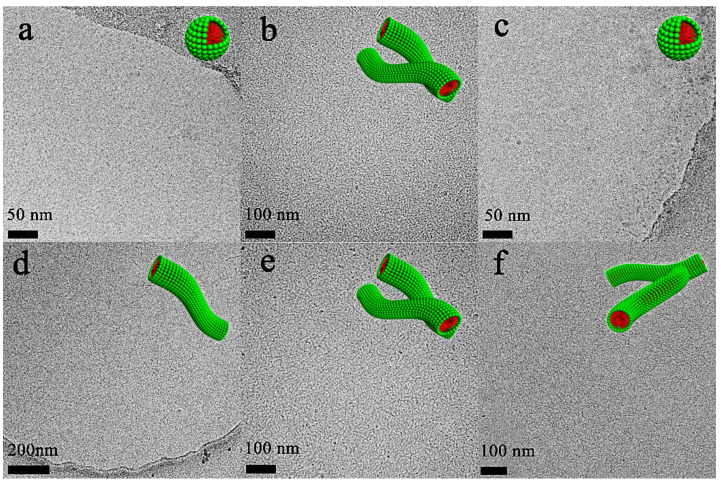
Cryo-TEM micrographs of the C_12_C_3_C_12_(SO_3_)_2_ and C_12_C_3_C_12_(SO_3_)_2_/CTAB aggregates. For 20.0 mM C_12_C_3_C_12_(SO_3_)_2_, the pH values are selected at 9.5 (**a**) and 6.7 (**b**); and for C_12_C_3_C_12_(SO_3_)_2_/CTAB mixtures at pH 6.7, the *C*_T_ values are determined at 0.1 mM (**c**), 1.0 mM (**d**), 20.0 mM (**e**) and 60.0 mM (**f**), respectively.

To have a deeper insight into the aggregation behaviors in each region determined above, DLS and Cryo-TEM were employed to characterize the aggregate morphology at pH 9.5 and 6.7. At high pH conditions (pH 9.5), a size distribution of aggregates with *R*_h_ of ~3 nm existed in the DLS result (Figure 2) and spherical micelles were observed in the Cryo-TEM image (Figure 3a). At neutral pH conditions (pH 6.7), a size distribution of aggregates with *R*_h_ of ~31 nm was found in the DLS result (Figure 2) and the Cryo-TEM results exhibited the entangled wormlike micelles which were responsible for the high viscosity (Figure 3b). All the results of C_12_C_3_C_12_(SO_3_)_2_ itself depending on pH described above signify that the aggregation transition underwent three regions with decreasing pH, i.e., spherical micelles, wormlike micelles, and precipitation.

**Figure 4:**
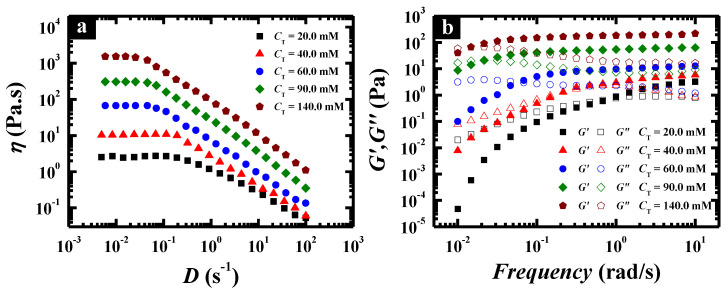
Steady (**a**) and dynamic (**b**) rheological properties of C_12_C_3_C_12_(SO_3_)_2_ solutions at different concentrations, and the total concentration is 20.0, 40.0, 60.0, 90.0 and 140.0 mM, respectively.

Steady and dynamic rheological measurements were performed to investigate the flow properties of the wormlike micellar solutions at different total concentrations, fixing the pH value at the optimal value (pH 6.7). The solutions behaved as a Newtonian fluid with low viscosity at low concentration, which was evidenced by the spherical micelles and separately wormlike micelles in Cryo-TEM images (Figure 3c,d). Above 20.0 mM, the solution changed to a non-Newtonian fluid with high viscosity and the zero-shear viscosity was significantly enhanced with increasing *C*_T_ (Figure 4a). When *C*_T_ increased to 140.0 mM, the zero-shear viscosity of the surfactant solution could reach as high as 1530 Pa·s, which was enhanced by nearly three orders of magnitude compared with that at *C*_T_ = 20.0 mM and six orders of magnitude compared with that in water. The dynamic rheological experiment (Figure 4b) indicates that the wormlike micelle solutions show a viscoelastic response above 20.0 mM, i.e., elastic behavior dominates at high frequencies (*G*′ > *G*″), whereas viscous behavior dominates at low frequencies (*G*′ < *G*″). In particular, for the solutions above 60.0 mM, *G*′ (ω) and *G*″ (*ω*) cross each other at a low frequency, i.e., a crossover frequency (*ω*_R_) in the range of 0.10 and 1.0 rad/s, of which the inverse is often used to determine the relaxation time (*τ*_R_). This is typical viscoelastic behavior shown by wormlike micellar solutions and the lower crossover frequency means a more remarkably viscoelastic fluid.

**Figure 5:**
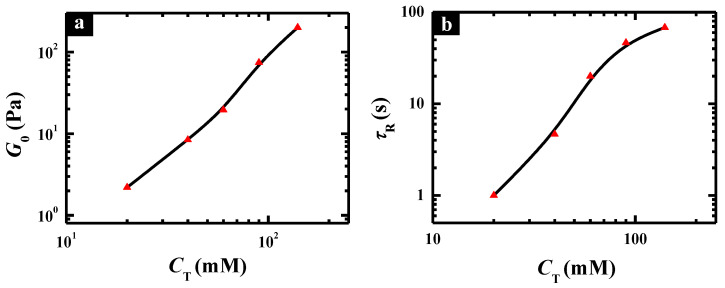
Variation of *G*_0_ (**a**) and *τ*_R_ (**b**) as a function of *C*_T_ for C_12_C_3_C_12_(SO_3_)_2_ solutions at pH 6.7.

To gain insight into the wormlike micelles formed in C_12_C_3_C_12_(SO_3_)_2_ aqueous solutions at pH 6.7, the rheological parameters, *G*_0_ and *τ*_R_, were further investigated as a function of *C*_T_, as shown in Figure 5. Here, *G*_0_ refers to the plateau modulus, i.e., the value of elastic modulus *G*′ reaching a plateau at high frequency. Generally, *G*_0_ measures the number of entanglements between wormlike micelles or the mesh size of the network structure, whereas *τ*_R_ is relevant to the contour length of the wormlike micelles. It can be observed that both the values of *G*_0_ and *τ*_R_ of C_12_C_3_C_12_(SO_3_)_2_ solutions showed a conspicuous monotonic increase with increasing *C*_T_, which meant both the number of entanglements and the contour length of the wormlike micelles were enlarged at a higher total concentration. This is broadly in accordance with the increasing viscosity obtained by steady rheological measurements.

The formation of wormlike micelles at low or neutral pH is definitely related to the special molecular structure of C_12_C_3_C_12_(SO_3_)_2_. Under alkaline conditions, the C_12_C_3_C_12_(SO_3_)_2_ molecules are completely deprotonated, so this surfactant acts as an anionic gemini surfactant bearing two negative charges. It is known that the short spacer of the cationic gemini surfactant will decrease the headgroup area, making it favorable for micellar growth. However, the stronger electrostatic repulsion and steric hindrance of the sulfonate groups result in the formation of spherical micelles for C_12_C_3_C_12_(SO_3_)_2_. Upon decreasing the pH, the added H^+^ bonds onto the tertiary amino to form quaternary ammonium cation, so that the anionic surfactant C_12_C_3_C_12_(SO_3_)_2_ will turn into a zwitterionic surfactant, like sulfobetaine gemini surfactant. The headgroup area of C_12_C_3_C_12_(SO_3_)_2_ is further decreased due to the electrostatic interaction between the sulfonate anion and quaternary ammonium cation, while the intermolecular hydrogen bond is formed which also draws the headgroup closer. Hence, the electrostatic attraction, the hydrogen bond and the short spacer are responsible for the micellar growth and the formation of wormlike micelles under low pH conditions.

Normally, the formation of wormlike micelles with high viscosity requires a long hydrophobic chain length, 16-carbon or longer [23,42,43,44,45]. Even though some surfactants have a 12-carbon-length tail, the high concentration is necessary for the high viscous solution [24,46,47]. Here the zero-shear viscosity of the C_12_C_3_C_12_(SO_3_)_2_ solution reached 300 Pa·s at 90.0 mM and even 1530 Pa·s at 140.0 mM, which was greatly improved compared with other surfactant systems with 12-carbon chain length and comparable to the surfactant systems with a longer chain length. It is assumed that both the intermolecular hydrogen bond and the multiple binding sites play a great role on the significant enhancement of viscosity. The hydrogen bonds occurring less than 5 Å could be strongly enhanced when more hydroxyl groups stay close to each other in the aggregates, due to the stronger hydrophobic interaction contributing to the excessive free energy to the end caps and inducing the formation of long micelles with a lower surface curvature. The contribution of the hydrogen groups to the high viscosity has been verified in previous work [34,48], in which the intermolecular hydrogen bonds are crucial for the pH-sensitive rheological behavior for *N*-cetyl-*N*,*N*-dihydroxyethylammonium bromide with OH groups, while the similar phenomenon can hardly be observed in *N*-cetyl-*N*,*N*-diethylammonium bromide without OH groups. The comparative studies on the viscosity of two cationic gemini surfactants, propanediyl-1,3-bis(dimethyldodecylammonium bromide) (C_12_C_3_C_12_Br_2_) and 2-hydroxyl-propanediyl-1,3-bis-(dimethyldodecylammonium bromide) (C_12_C_3_(OH)C_12_Br_2_) also provide the evidence for the importance of the OH group for viscosity enhancement.

The influence of pH on the aggregate transition in C_12_C_3_C_12_(SO_3_)_2_/CTAB mixtures. It is well known that a long chain length is beneficial for the increase in viscosity of surfactant aqueous solutions and mixing cationic/anionic surfactants can weaken the electrostatic interaction between charged headgroups and in turn promote micelle growth and finally form wormlike micelles. Herein, CTAB was selected to be mixed with C_12_C_3_C_12_(SO_3_)_2_, and the influence of pH on their aggregation behavior is investigated. The molar fraction of C_12_C_3_C_12_(SO_3_)_2_ (*X*_g_) is fixed at 0.30 and 0.70 as the representative condition.

**Figure 6:**
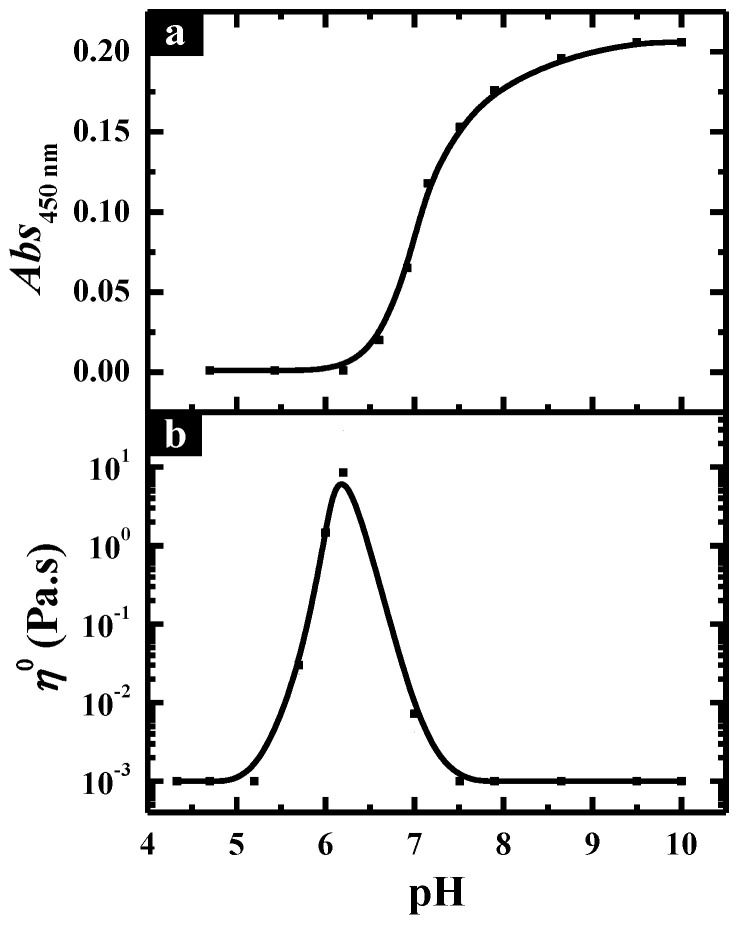
(**a**) Turbidity and (**b**) zero-shear viscosity *η*^o^ of C_12_C_3_C_12_(SO_3_)_2_/CTAB solutions plotted against pH at *X*_g_ = 0.30 and *C*_T_ = 20.0 mM.

Turbidity and rheology were also employed to characterize the aggregate transition in C_12_C_3_C_12_(SO_3_)_2_/CTAB mixtures through changing the pH at *X*_g_ = 0.30 and *C*_T_ = 20.0 mM as shown in Figure 6a. Upon decreasing pH, the turbidity of the solutions dropped rapidly until it reached a considerably low value at pH 6.5, below which the turbidity remained unchanged. Figure 6b is the variation of zero-shear viscosity plotted as a function of pH. Correspondingly, the viscosity of C_12_C_3_C_12_(SO_3_)_2_/CTAB mixtures was similar to that of water at high pH values and began to increase at pH 7.5 until a maximum at pH 6.1, below which the viscosity returned to a low value again. In the pH range of 5.0 and 7.5, the non-Newtonian behavior occurred, and shear thinning was observed, signifying the formation of the entangled wormlike micelles. Considering the formation of vesicles at pH 9.2 reported in our previous work [41] and the high turbidity low viscosity after mixing with CTAB, the changes in the turbidity and viscosity with decreasing pH should be corresponding to the transition process from vesicle to wormlike micelle.

**Figure 7:**
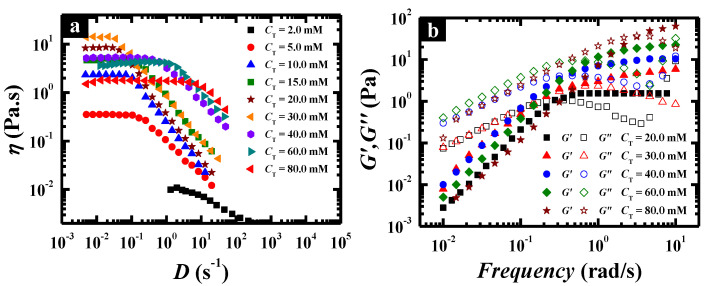
Rheological properties of C_12_C_3_C_12_(SO_3_)_2_/CTAB mixtures at *X*_g_ = 0.30 and pH 6.7. (**a**) Steady shear viscosity. (**b**) Elastic modulus *G*′ and viscous modulus *G*″ as functions of angular frequency *ω*.

To have a deeper insight into the wormlike formation at pH 6.7, steady/dynamic rheological measurements, DLS and Cryo-TEM were employed to characterize the aggregates at different concentrations. Figure 7a depicts the steady viscosity of C_12_C_3_C_12_(SO_3_)_2_/CTAB mixtures at *X*_g_ = 0.30 and pH 6.7. Below 2.0 mM, the viscosity of the mixtures was like that of water and Newtonian flow behavior was observed. The spherical micelles existed at 0.10 mM (Figure 3c) and elongated to long wormlike micelles of a few micrometers at 0.10 mM (Figure 3d), indicating that the micellar growth had already occurred, but the elongated micelles hardly entangled with each other at the low concentration, so that the viscosity was as low as that of water. Above 2.0 mM, the Newtonian behavior occurred at low shear rates only and shear thinning was observed at large shear rates. Interestingly, a remarkable increase in the zero-shear viscosity occurred as the total concentration varied from 2.0 mM to 30.0 mM, afterwards there was an unexpectedly slow descent until 80.0 mM. The maximum at 30.0 mM was about 20 Pa·s, nearly four orders of magnitude higher than that of water and one order of magnitude higher than that of single C_12_C_3_C_12_(SO_3_)_2_. Undoubtedly, this increase in viscosity below 30.0 mM contributed to the formation of more and more entangled wormlike micelles verified by Figure 3e. The addition of a large amount of cationic surfactant CTAB weakened the electrostatic interaction between the charged headgroups and enhanced the growth of micelles.

Above 30.0 mM, the zero-shear viscosity began to decrease with increasing *C*_T_, which might be attributed to the formation of branched micelles. There has been some evidence to support our prediction, which appeared in the mixtures of sodium oleate (NaOL) and octyltrimethylammonium bromide (C8TAB) [42] and dodecyltriethylammonium bromide (DTEAB)/sodium dodecylsulfate (SDS) [49]. Here the dense and crosslinked wormlike micelles with many junctions between each one was clearly observed by Cryo-TEM in Figure 3f. The higher concentration would increase the energy cost for the formation of hemispherical end-caps of the cylindrical micelles, in which case the end-cap energy can be minimized by fusing the free ends into the cylindrical part of its own or other micelles, thus developing into branched wormlike micelles.

To further understand the nature of branched wormlike micelles, dynamic rheological measurements were performed on catanionic mixtures with varying *C*_T_. Figure 7b shows the variation of elastic and viscous modulus, *G*′ and *G*″, as a function of frequency. All the solutions at different concentrations showed typical viscoelastic behavior, and there was a decrease in the crossover frequency (*ω*_R_) below *C*_T_ = 30.0 mM followed by an increase above *C*_T_ = 30.0 mM. However, the plateau modulus kept rising with increasing *C*_T_ although there was a maximum of viscosity. Hence, corresponding to the transition from entangled wormlike micelles to branched ones, the entanglements between wormlike micelles or the mesh size of the network structure continued increasing, whereas the contour length of the wormlike micelles rose first and then began to decrease.

**Figure 8:**
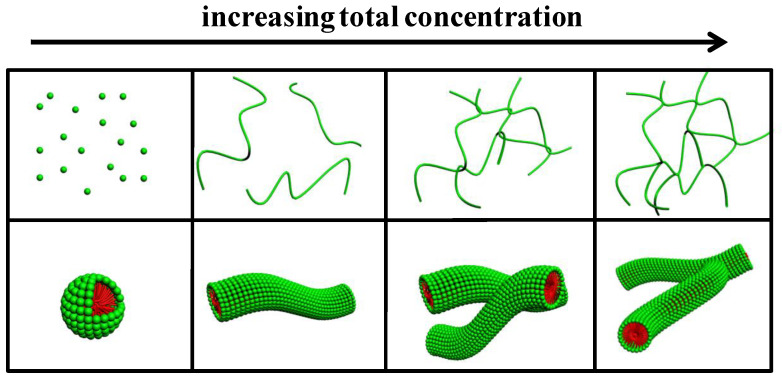
Model of aggregate transition with increasing concentration at *X*_g_ = 0.30 and pH 6.7.

Combining all the measurements in the C_12_C_3_C_12_(SO_3_)_2_/CTAB mixtures at *X*_g_ = 0.30, the possible models of the aggregate transitions are illustrated in Figure 8. The C_12_C_3_C_12_(SO_3_)_2_/CTAB mixtures started to self-assemble into spherical micelles above *C*_T_ = 0.06 mM. With increasing *C*_T_, micellar growth occurred and spherical micelles transitioned into elongated wormlike micelles, but the solution viscosity was considerably low under these conditions, due to few entanglements occurring between each other. When *C*_T_ reached a threshold value, ~2.0 mM (C*), the wormlike micelles began to entangle into a network structure, in which region the mixed solutions behaved as a non-Newton fluid and the viscosity kept rising with increasing *C*_T_. Further increasing the *C*_T_ above 30.0 mM, the long entangled wormlike micelles transferred into the branched ones, which were responsible for the viscosity declination. Therefore, by increasing the total concentration at *X*_g_ = 0.30 and pH 6.7, the mixed aggregates underwent three stages of transition process, i.e., spherical micelles → elongated micelles → entangled wormlike micelles → branched wormlike micelles.

**Figure 9:**
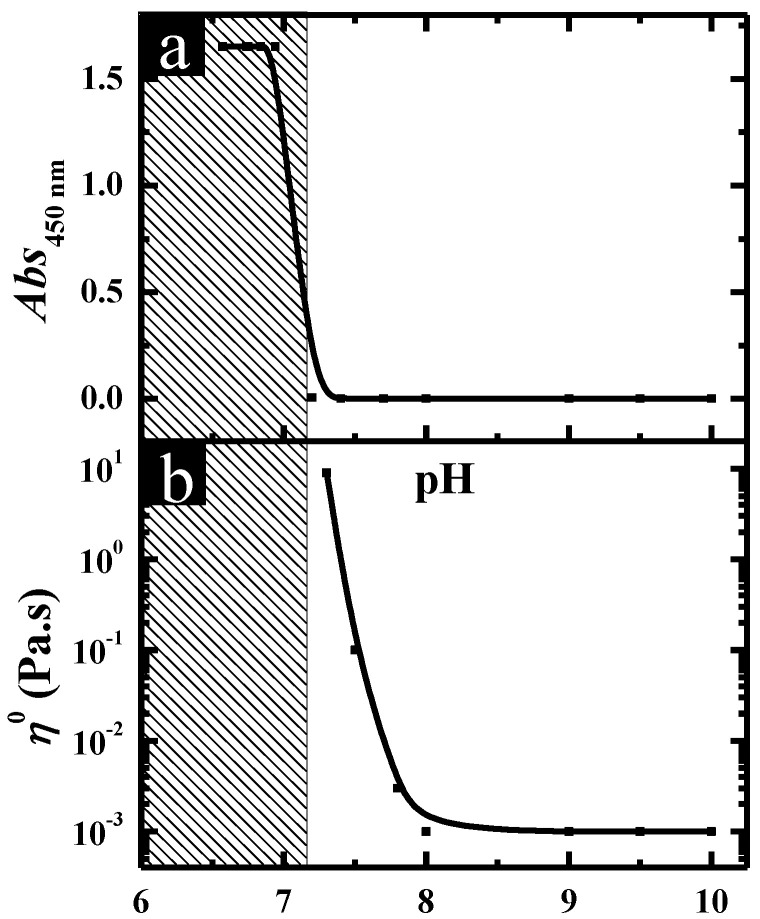
(**a**) Turbidity and (**b**) zero-shear viscosity *η*^o^ of C_12_C_3_C_12_(SO_3_)_2_/CTAB solutions plotted against pH at *X*_g_ = 0.70 and *C*_T_ = 20.0 mM.

**Figure 10:**
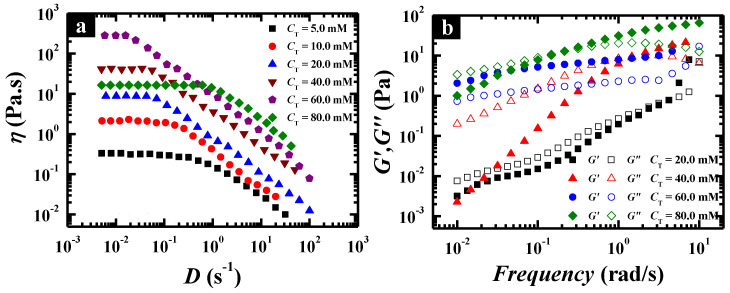
Rheological properties of C_12_C_3_C_12_(SO_3_)_2_/CTAB mixtures at *X*_g_ = 0.70. (**a**) Steady shear viscosity, (**b**) elastic modulus *G*′ and viscous modulus *G*″ as functions of angular frequency *ω*.

According to the results above, it is possible to improve the rheological properties of a C_12_C_3_C_12_(SO_3_)_2_/CTAB mixed solution through adjusting the molar ratio of the two components, so the pH-induced aggregate transition at *X*_g_ = 0.70 was also investigated by turbidity and steady rheological measurements as shown in Figure 9. Upon changing the pH from 10.0 to 6.0, the turbidity displayed a small value that was close to zero and almost had no change until pH 7.2, following which, precipitation takes place (Figure 9a). In the dissolved state, the zero-shear viscosity started to be enhanced from pH 7.5 (Figure 9b), corresponding to the transition process from spherical micelle to wormlike micelle. Then fixing the pH at 7.5, the steady and dynamic rheological measurements were carried out for the mixed solution with different total concentrations (Figure 10). Similar to *X*_g_ = 0.30, the decrease in viscosity induced by the higher concentration can be also observed at a large molar fraction, which should be ascribed to the formation of branched wormlike micelles. However, the viscosity maximum occurred at *X*_g_ = 0.70 and *C*_T_ = 60.0 mM approached about 280 Pa·s, which was much higher than that at *X*_g_ = 0.30 and the same total concentration. It suggests that the addition of more C_12_C_3_C_12_(SO_3_)_2_ molecules can produce a stronger intermolecular hydrogen-bonding interaction, facilitating the formation of a viscous solution, but the weakened charges of the headgroups in the catanionic mixed system would greatly induce the entanglement of many wormlike micelles and even fusion to each other, thus resulting in branched microstructures and the decline of viscosity.

## 4. Conclusions

pH-responsive wormlike micelle systems were constructed both in single gemini surfactant C_12_C_3_C_12_(SO_3_)_2_ solutions and in C_12_C_3_C_12_(SO_3_)_2_/CTAB mixtures. In C_12_C_3_C_12_(SO_3_)_2_ solutions, the rheological properties were greatly improved due to the transition from spherical micelles to entangled wormlike micelles upon decreasing the pH, and its viscoelastic properties were greatly enhanced compared with other C-12 hydrophobic surfactant systems. There are three reasons for the significant micellar growth and entanglement, i.e., short spacer, sulfobetaine zwitterionic headgroup and hydrogen-bonding sites. Especially at 140 mM and pH 6.7, the mixture showed high viscoelasticity, and the maximum of the zero-shear viscosity reached 1530 Pa·s, which is ca. six orders of magnitude larger than that of water. In C_12_C_3_C_12_(SO_3_)_2_/CTAB mixtures, a similar transition process can be triggered by pH, and a more complex aggregation behavior, monomer→spherical micelles→elongated micelles→entangled micelles→branched micelles, was monitored upon increasing total concentration. Although the mixtures could not approach the viscosity maximum appearing in the C_12_C_3_C_12_(SO_3_)_2_ solution, the CTAB addition was more favorable for viscosity enhancement in the wormlike-micelle region. The weakened charges of the headgroups in the catanionic mixed system minimized the micellar spontaneous curvature and enhanced the intermolecular hydrogen-bonding interaction between C_12_C_3_C_12_(SO_3_)_2_, facilitating the formation of a viscous solution, while greatly induced entanglement and even the fusion of wormlike micelles, thus resulting in branched microstructures and a decline in viscosity. This work may make us further understand the unique superiority of gemini surfactant in improving rheological properties and provide an efficient way to enhance the solution’s viscoelastic properties.

## Data Availability

Data is contained within the article.

## Sample Availability

Samples of the compounds C_12_C_3_C_12_(SO_3_)_2_ and CTAB are available from the authors.
